# Convergent and Divergent Age Patterning of Gut Microbiota Diversity in Humans and Nonhuman Primates

**DOI:** 10.1128/msystems.01512-21

**Published:** 2022-06-27

**Authors:** Jianan Sang, Daohua Zhuang, Tao Zhang, Qunfu Wu, Jiangkun Yu, Zhigang Zhang

**Affiliations:** a State Key Laboratory for Conservation and Utilization of Bio-Resources in Yunnan, School of Life Sciences, Yunnan University, Kunming, Yunnan, China; b State Key Laboratory of Genetic Resources and Evolution, Laboratory of Evolutionary & Functional Genomics, Kunming Institute of Zoology, Chinese Academy of Sciences, Kunming, Yunnan, China; University of Southampton

**Keywords:** humans, nonhuman primates, aging, gut microbiota, plasma metabolomics

## Abstract

The gut microbiome has significant effects on healthy aging and aging-related diseases, whether in humans or nonhuman primates. However, little is known about the divergence and convergence of gut microbial diversity between humans and nonhuman primates during aging, which limits their applicability for studying the gut microbiome’s role in human health and aging. Here, we performed 16S rRNA gene sequencing analysis for captive rhesus macaques (Macaca mulatta) and compared this data set with other freely available gut microbial data sets containing four human populations (Chinese, Japanese, Italian, and British) and two nonhuman primates (wild lemurs [Lemur catta] and wild chimpanzees [Pan troglodytes]). Based on the consistent V4 region of the 16S rRNA gene, beta diversity analysis suggested significantly separated gut microbial communities associated with host backgrounds of seven host groups, but within each group, significant gut microbial divergences were observed, and indicator bacterial genera were identified as associated with aging. We further discovered six common anti-inflammatory gut bacteria (*Prevotellamassilia*, *Prevotella, Gemmiger*, *Coprococcus*, *Faecalibacterium*, and *Roseburia*) that had butyrate-producing potentials suggested by pangenomic analysis and that showed similar dynamic changes in at least two selected host groups during aging, independent of distinct host backgrounds. Finally, we found striking age-related changes in 66 plasma metabolites in macaques. Two highly changed metabolites, hydroxyproline and leucine, enriched in adult macaques were significantly and positively correlated with *Prevotella* and *Prevotellamassilia*. Furthermore, genus-level pangenome analysis suggested that those six common indicator bacteria can synthesize leucine and arginine as hydroxyproline and proline precursors in both humans and macaques.

**IMPORTANCE** This study provides the first comprehensive investigation of age patterning of gut microbiota of four human populations and three nonhuman primates and found that *Prevotellamassilia*, *Prevotella*, *Gemmiger*, *Coprococcus*, *Faecalibacterium*, and *Roseburia* may be common antiaging microbial markers in both humans and nonhuman primates due to their potential metabolic capabilities for host health benefits. Our results also provide key support for using macaques as animal models in studies of the gut microbiome’s role during human aging.

## INTRODUCTION

Aging, which is regulated by both genetic and environmental factors (1, 2), is accompanied by declines in sensory, motor, and cognitive functions, ultimately leading to death (3). A general decline in the diversity of individuals’ core gut microbiota as they age (4, 5) is related to increases in frailty, immunosenescence, and chronic systemic inflammation (6). Abundant evidence suggests that the gut microbiome might be a modulator of healthy aging, and different human populations across the world have distinct aging-associated gut microbes due to the effects of multiple complex factors, including host genetics, gender, diets, habitats, lifestyles, and even different 16S rRNA gene sequencing strategies (7). Therefore, there is a clear need for validated animal models to simulate gut microbial changes in humans during aging, identify microbial biomarkers to study human aging-related diseases, and develop effective interventions.

Numerous pieces of experimental evidence suggest the causal relationships between gut microbiota and aging with model organisms. The African turquoise killifish, Nothobranchius furzeri, can be rejuvenated after transplantation with gut microbiota of younger individuals, which prevents the decline of microbial diversity during host aging (8). The polysaccharide colanic acid secreted by Escherichia coli can extend the life span of Caenorhabditis elegans via action on ATFS-1, a host UPR^mt^-responsive transcription factor (9). In addition, the gut bacterium *Acetobacter* can promote aging-related intestinal dysfunction and shorten the life span of *Drosophila*, while low doses of oxidants during development can selectively eliminate *Acetobacter* and increase its life span (10). These causal relationships between the gut microbiota and the aging process in animal models can improve the understanding of the gut microbiome's effects on healthy human aging and aging-related diseases. Furthermore, studies of our primate relatives, specifically with macaques, are highly valuable as they add the ability to assess impacts on neurodegenerative diseases (11). Aging macaques naturally develop cognitive deficits, amyloid plaques, and tau pathology with the same qualitative pattern and sequence as in humans (12). The anti-inflammatory bacteria *Blautia*, *Coprococcus*, and *Roseburia* are significantly less abundant in the fecal microbial community of Parkinson’s disease patients than in controls (13). Similarly, A53T transgenic macaques with early Parkinsonian symptoms showed obvious differences in gut microbiota and metabolites from control macaques, with higher diversity of gut microbiota and significantly higher abundance of *Sybergistetes*, *Akkermansia*, and Eggerthella lenta, but lower abundance of *Prevotella* (14). Yet, these findings suggested that the macaque cannot fully simulate the human gut microbial community structure, although it has great advantages as an animal model. Recently, a study showed that the gut microbiota of human and nonhuman primates have similar butyrate-producing pathways (15). Reductions in butyrate and butyrate-producing bacteria may lead to related diseases (i.e., Crohn’s disease) (16), and butyrate supplements could attenuate negative effects of aging (17). Thus, it is essential to explore potentially common antiaging microbial markers (i.e., butyrate-producing bacteria) by much more investigation of human populations and nonhuman primates.

Here, we first investigated age patterning in gut microbial diversity of 49 macaques by 16S rRNA gene sequencing analysis. We further explored the divergence and convergence of aging-associated gut microbial diversities in humans and nonhuman primates by integrating freely available data sets from two long-living human populations (Chinese and Italian) (18, 19), two other healthy populations (Japanese and British) (20) (American Gut Project; http://americangut.org), and two additional wild primate relatives (lemurs and chimpanzees) (21, 22). Finally, we also analyzed differences in plasma metabolites between the young and elderly macaques and explored metabolome-microbiome associations to probe potential antiaging gut microbial markers.

## RESULTS

Fresh fecal samples were collected from 18 adult and 31 elderly macaques (matched human ages of 13.5 ± 1.5 and 60 ± 9.53 years old, respectively) (see details from Table S1 in the supplemental material). Fecal microbiota of the 49 macaques were determined by high-throughput sequencing of the V3/V4 region of the bacterial 16S rRNA gene (Materials and Methods). To discover general age patterning of gut microbial diversity of humans and nonhuman primates, we collected previously published 16S rRNA gene data sets of the gut microbiota of 189 adult (20 to 40 years old) and 197 elderly (50 to 80 years old) individuals from 88 Chinese (13 adults and 75 elderly), 26 Italian (13 adults and 13 elderly), 208 Japanese (135 adults and 73 elderly), and 64 British (28 adults and 36 elderly) subjects. We also collected data sets from two additional wild primate relatives, including 13 lemur adults (matched human age, 15 ± 3.65 years) and 13 lemur elderly (matched human age, 30 ± 1.92 years) and 314 chimpanzee adults (matched human age, 23 ± 18.46 years) and 137 chimpanzee elderly (matched human age, 53 ± 18.52 years) (see other details in Table S1). We chose the V4 region of 16S rRNA gene for further analysis because it can cover all data sets and overcome the biases from inconsistent regions of 16S rRNA gene sequencing from different data sets. After quality filtering of merged data sets, we obtained 79,967,252 high-quality tags that were clustered into 8,363 zero-radius operational taxonomic units (ZOTUs) according to 100% sequence identity using USEARCH software (version 11.0.667 i86linux64) (23). To keep many more samples, the reads were subsampled to a consistent sequence depth of 1,347 reads per sample according to the minimum number of reads. The remaining 6,732 ZOTUs were used for further analysis.

10.1128/msystems.01512-21.3 TABLE S1 Sample information. Download Table S1, XLSX file, 0.1 MB. Copyright © 2022 Sang et al. 2022 Sang et al. https://creativecommons.org/licenses/by/4.0/ This content is distributed under the terms of the Creative Commons Attribution 4.0 International license.

### Changes in gut microbial composition of seven groups during aging.

**FIG 1:**
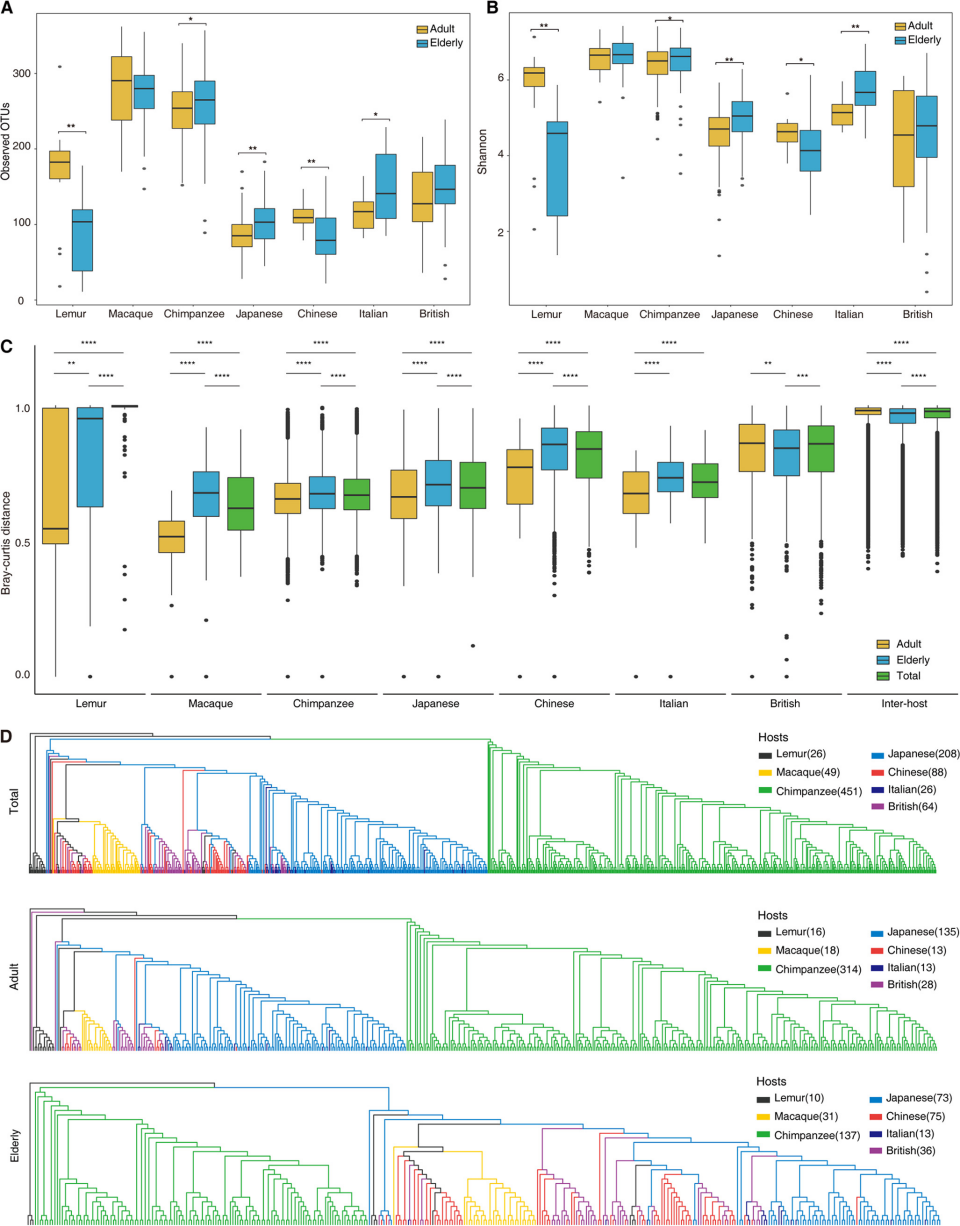
Alpha and beta diversity measurements. (A and B) Difference in alpha diversity between adults and the elderly by richness (observed OTU) and Shannon indices. By Wilcoxon’s test, *, *P* < 0.05, and **, *P* < 0.01. (C) Difference in Bray-Curtis distance within host groups and between host groups. By Wilcoxon’s test, **, *P* < 0.01, ***, *P* < 0.001, and ****, *P* < 0.0001. (D) UPGMA trees based on Bray-Curtis distance of total individuals, adults, and the elderly, respectively.

We found significant alternations of alpha diversity (Richness and Shannon) of gut microbiota between adults and elderly of five of seven host groups, except macaques and British subjects (*, *P* < 0.05, and **, *P* < 0.01, by Wilcoxon test) (Fig. 1A and 1B). Compared with adults, significant declines in alpha diversity were observed only in elderly individuals of the lemur (*P* < 0.01) and Chinese (*P* < 0.01) groups. In contrast, gut microbial diversity was significantly higher in the elderly than adults in the chimpanzee (*P* < 0.05), Japanese (*P* < 0.01), and Italian (*P* < 0.01) groups.

Principal-coordinate analysis (PCoA) plotting based on Bray-Curtis distances showed significant separation (adjusted *R*^2^ = 0.218, *P* = 0.001) of gut microbial community structures of seven host groups (Fig. S1A), consistent with the results implicated by UPGMA (Unweighted Pair Group Method with Arithmetic means) trees (Fig. 1D), which were mostly determined by host backgrounds, including host genetic factors and/or diet, but not by disparity in age (Fig. 1C; Fig. S1A). The divergence of host genetics solely cannot explain gut microbial variations across host groups because chimpanzees are the closest living relatives of humans (24), but the gut microbiota of the macaque was the most closely related to humans but not that of chimpanzees (Fig. 1D). Furthermore, we also discovered significant differences of gut microbial communities between adults and the elderly for each host group (Fig. 1C; Fig. S1B to S1F), suggesting the existence of aging-associated microbial markers in both humans and nonhuman primates.

10.1128/msystems.01512-21.1 FIG S1 Principal-coordinate analysis (PCoA) plotting based on Bray-Curtis distances of gut microbial communities across hosts (A) and between adults and the elderly (B to H). Download FIG S1, EPS file, 2.5 MB. Copyright © 2022 Sang et al. 2022 Sang et al. https://creativecommons.org/licenses/by/4.0/ This content is distributed under the terms of the Creative Commons Attribution 4.0 International license.

### Age patterning of gut microbial compositions in humans and nonhuman primates.

**FIG 2:**
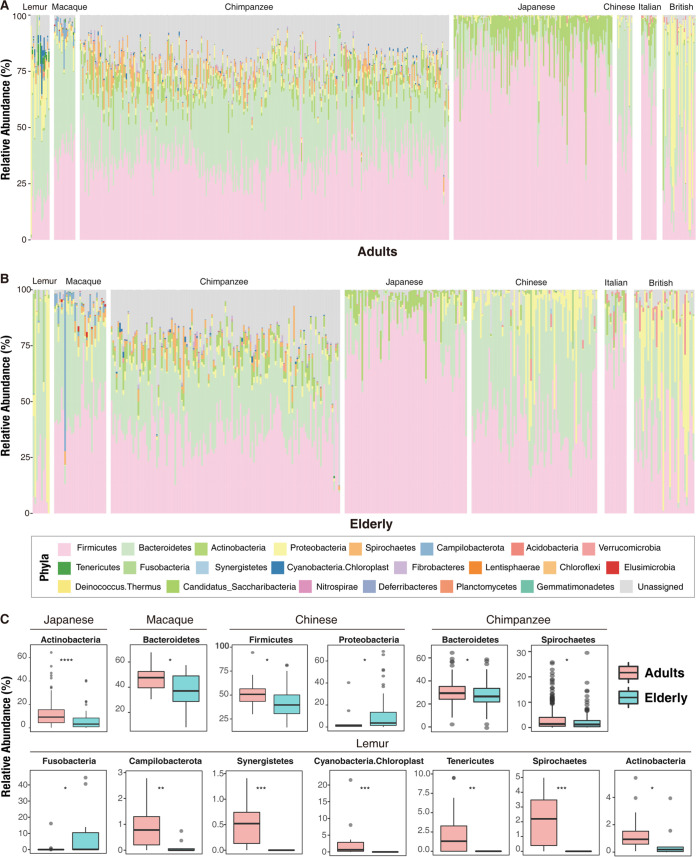
Relative abundances of 22 gut bacterial phyla. (A and B) Relative abundance of gut bacterial phyla in seven groups of adult/elderly individuals; (C) phyla with significant differences among host groups during aging.

To identify convergent and divergent changes of the gut microbiota in humans and nonhuman primates, we examined age-related changes in relative abundance of total 22 gut bacterial phyla (14 in lemurs, 16 in macaques, and 18 in chimpanzees and 10 in Chinese, 8 in Italian, 8 in Japanese, and 10 in British subjects) (Fig. 2A). The *Firmicutes* and *Bacteroidetes* were the top two dominant gut bacterial phyla despite whether the hosts were humans or nonhuman primates, with the exception of Japanese subjects. The top two phyla in the gut of Japanese subjects are *Firmicutes* (mean ± standard deviation [SD], 80.12% ± 13.43%) and *Actinobacteria* (9.71% ± 10.41%). The *Firmicutes* were the most dominant phylum in the Italian subjects (74.32% ± 12.54%), macaques (43.51% ± 9.80%), chimpanzees (36.61% ± 9.30%), and British subjects (33.61% ± 20.34%), respectively. However, the *Bacteroidetes* were the most abundant phylum in Chinese subjects (43.38% ± 19.90%) and lemurs (39.73% ± 22.45%). In addition, *Bacteroidetes* were also significantly abundant in the Chinese group (Fig. S2B). The *Firmicutes* and *Actinobacteria* in Japanese subjects were significantly higher than in other hosts (Fig. S2B). These results suggest there are fundamental differences of dominant gut bacterial compositions across different host groups, possibly reflecting the impacts of host backgrounds. During aging, aging-associated shifts of dominant or other rare bacterial phyla were obviously observed in specific host groups (Fig. 2C). We first found significant reduction (*P* < 0.05) of the *Bacteroidetes* phylum in the elderly chimpanzees and macaques. Similarly, a significant decrease (*P* < 0.05) in the *Firmicutes* phylum was observed in the elderly Chinese. For other rare bacterial phyla, there were significant increases in *Proteobacteria* and *Fusobacteria* in the elderly Chinese and lemurs, respectively. The decrease in *Actinobacteria* was found in both the Japanese and lemur elderly. These findings indicated that the changes of specific gut bacterial compositions might have essential impacts on host aging.

**FIG 3:**
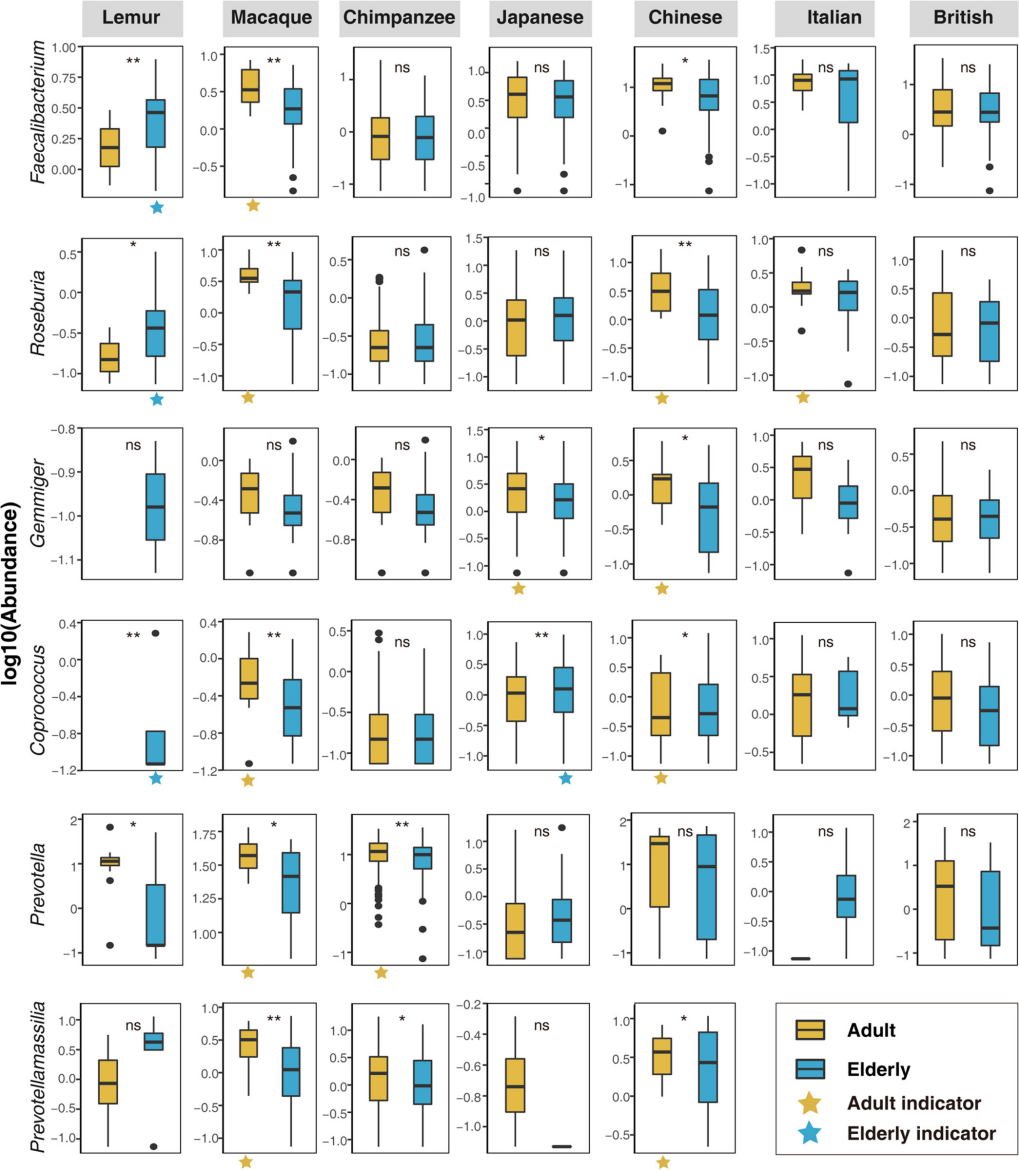
Age dynamics of six common indicator genera. Shown is the abundance of genera by age groups (defined in the text) of seven data sets: lemurs, macaques, and chimpanzees and Japanese, Chinese, Italian, and British subjects. Indicator analysis used the “indval” function in the “labdsv” package in R. By Wilcoxon’s test, ns, not significant, *, *P* < 0.05, and **, *P* < 0.01. Yellow stars indicate the presence of indicator bacteria in adults, and blue stars indicate the presence of indicator bacteria in the elderly.

Based on its presence, absence, or abundance, an indicator species can signal a change in the biological condition of a particular ecosystem and thus may be used as a proxy to diagnose the health of an ecosystem (25). Here, we applied the “indval” function of “labdsv” packages (26) in R with a *P* value lower than 0.05 as statistical significance to identify potential aging-related gut microbial markers or indicator taxa of each host group. We used a genus-level OTU table to identify indicator bacterial genera associated with aging. Consequently, we identified 31, 14, 11, 11, 3, 17, and 6 indicator bacterial genera that can differentiate the differences of gut microbiota between adults and elderly of the lemur, macaque, chimpanzee, Chinese, Italian, Japanese, and British groups, respectively (Table S2). As expected, six common indicator bacterial genera were found, including *Faecalibacterium*, *Roseburia*, *Gemmiger*, and *Coprococcus* from the *Firmicutes* phylum and *Prevotella* and *Prevotellamassilia* from the *Bacteroidetes* phylum, which showed similar age dynamics around at least two selected host groups (Fig. 3). *Faecalibacterium* and *Roseburia* were significantly more abundant in the elderly lemurs than adults and yet showed converse patterns in both macaques and Chinese subjects. *Gemmiger* declined significantly during aging only in both Japanese and Chinese subjects. *Coprococcus* had significant decrease in the elderly macaques, and a decrease was shown in both the Italian and British groups. In contrast, *Coprococcus* was significantly increased in elderly lemurs and Japanese and Chinese subjects. *Prevotella* significantly declined with increases in age in lemurs, macaques, and chimpanzees. Similarly, *Prevotellamassilia* significantly declined in the elderly macaques, chimpanzees, and Chinese subjects.

10.1128/msystems.01512-21.4 TABLE S2 Identification of indicator bacterial genera differentiating adults and elderly within host group. Download Table S2, XLSX file, 0.02 MB. Copyright © 2022 Sang et al. 2022 Sang et al. https://creativecommons.org/licenses/by/4.0/ This content is distributed under the terms of the Creative Commons Attribution 4.0 International license.

### Predicted functional characteristics of aging-associated indicator bacteria.

**FIG 4:**
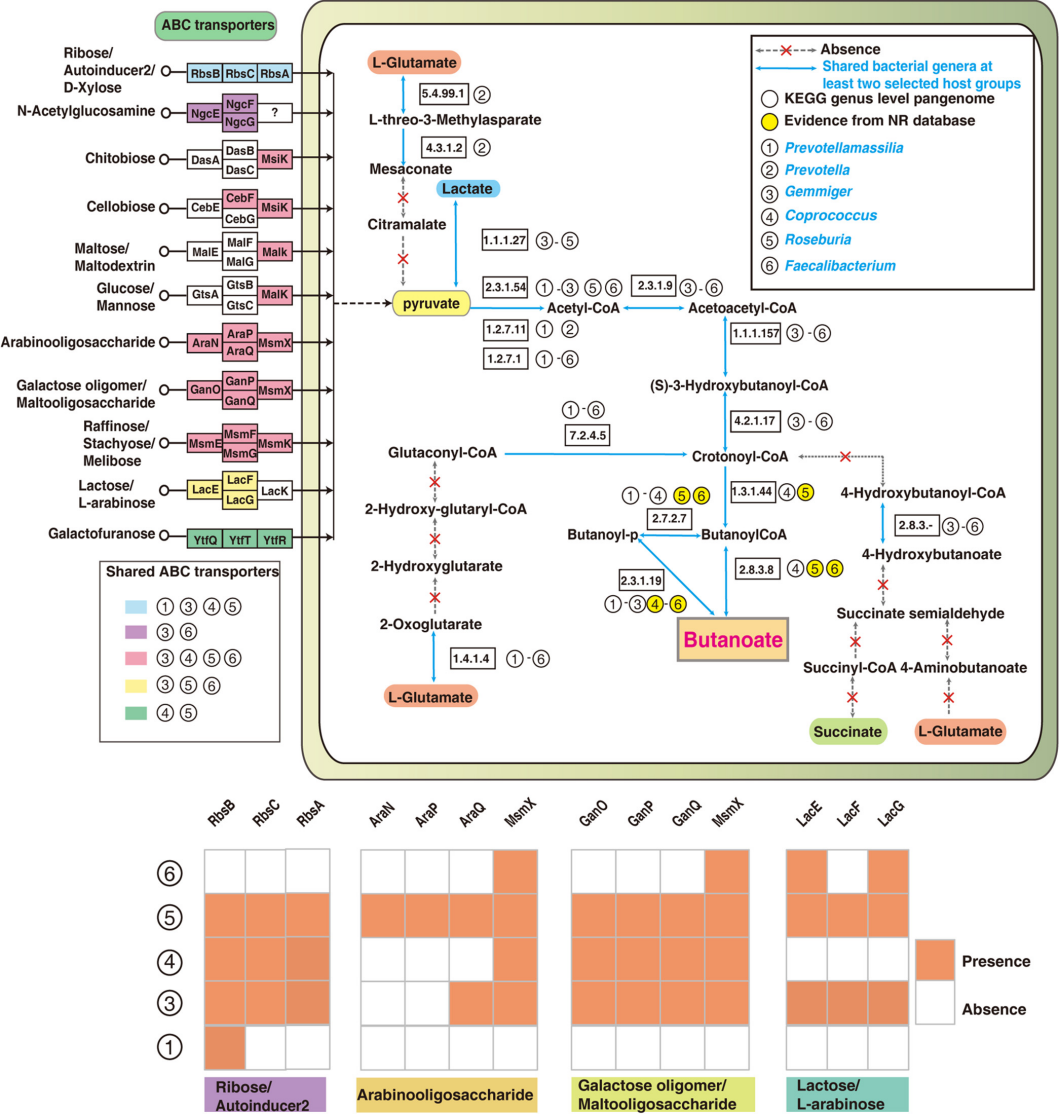
Predictions of butyrate-producing pathways based on pangenomic analysis of aging-associated indicator bacteria. All metabolic pathways were reconstructed by referring to pangenome pathway maps (map02010, map00660, map00620, map00310, map00650, and map00250) in the KEGG genome database. Circled numbers 1 to 6 represent six common aging-associated indicator bacteria, and those enzymes involved in all pathways were annotated using the KEGG and NCBI nr databases. The white and yellow circles indicate annotated results by the KEGG and nr databases, respectively. Colored boxes indicate ABC transporters shared by indicator bacteria. Circled genera 1 and 3 to 6 harbor different signal peptides as indicated by the orange blocks.

To obtain a functional view of the indicators potentially contributing to butyrate production, we performed pangenome predictions using KEGG tools (https://www.genome.jp/kegg/) and BLAST searches of their protein sequences in the NCBI nr database (https://ncbi.nlm.nih.gov/) (Table S3). We detected six indicator genera that can be annotated by the KEGG database: *Prevotellamassilia*, *Prevotella*, *Gemmiger*, *Coprococcus*, *Roseburia*, and *Faecalibacterium*. Predicted functional abilities of the indicator genera include abilities to utilize monosaccharides (ribose/autoinducer 2/d-xylose, glucose/mannose, galactofuranose) or oligosaccharides (*N*-acetylglucosamine, chitobiose, cellobiose, maltose/maltodextrin, arabinoligosaccharide, galactose oligomer/maltooligosaccharide, raffinose/stachyose/melibiose, lactose/l-arabinose) through ABC transporters (Fig. 4). Our results found that these indicator genera have distinct carbohydrate utilization abilities. Of these genera, only *Gemmige*r and *Faecalibacterium* can use *N*-acetylglucosamine, but *Faecalibacterium* lacks the ability to use ribose/autoinducer 2/d-xylose and galactofuranose. In addition, *Coprococcus* cannot use *N*-acetylglucosamine and lactose/l-arabinose. KEGG prediction reveals a lack of monosaccharide and oligosaccharide utilization in *Prevotella*. Streptococcus, *Coprococcus*, *Roseburia*, *Faecalibacterium*, and *Phascolarctobacterium* all harbor at least one signal peptide for transporting these oligosaccharides, but their transmembrane transporters slightly differ. Most enzymes involved in the butyrate-producing pathway can be expressed by six indicator bacterial genera according to the KEGG predictions and NCBI protein BLAST searches (Fig. 4). Six indicator genera can first generate butanoyl-p through butyrate kinase (EC 2.7.2.7) and then butanoate through phosphate butyryltransferase (EC 2.3.1.19). *Coprococcus*, *Roseburia,* and *Faecalibacterium* can directly generate butanoate through acetate coenzyme A (CoA)-transferase (EC 2.8.3.8).

10.1128/msystems.01512-21.5 TABLE S3 Prediction of key butyrate-producing enzymes of *Roseburia*, *Facalibacterium*, and *Coprococcus* based on the annotation of Nr database. Download Table S3, XLSX file, 0.02 MB. Copyright © 2022 Sang et al. 2022 Sang et al. https://creativecommons.org/licenses/by/4.0/ This content is distributed under the terms of the Creative Commons Attribution 4.0 International license.

### Age-associated shifts of plasma metabolomes in macaques.

**FIG 5:**
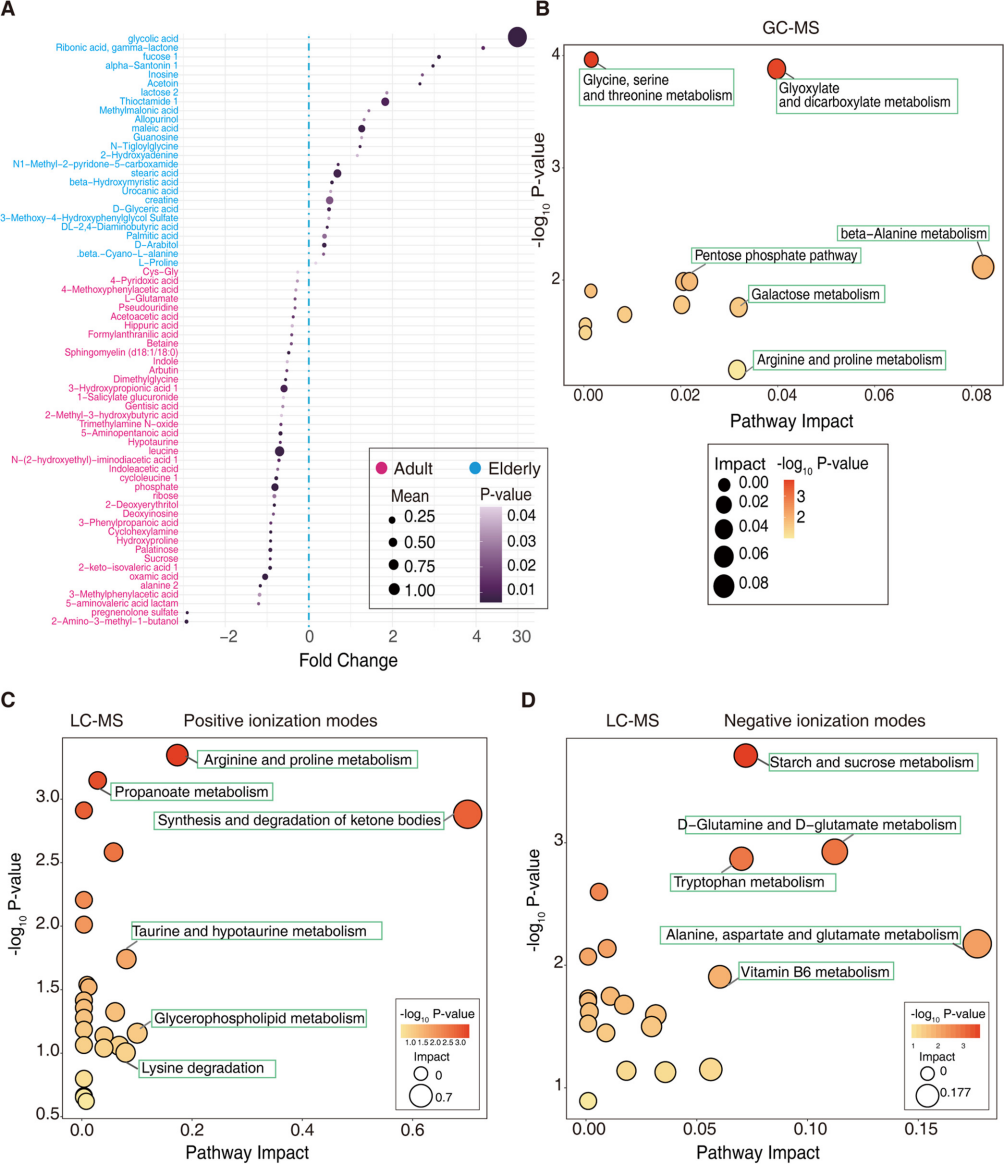
Differential plasma metabolites and pathway enrichment in macaques. (A) Sizes of dots represent fold change values [log_2_ (mean *E*/mean *Y*)] of enrichment in elderly and adult macaques (fold changes of >0 and <0, respectively), and their colors represent magnitudes of *P* values. Only *P* values of <0.05 are shown. (B to D) Pathway enrichment results of differential plasma metabolites of elderly versus adult macaques by GC-MS (B) and LC-MS (C and D), representing positive- and negative-ionization modes, respectively. Each dot represents a metabolic pathway, where the size indicates the pathway’s impact in the topology analysis, and the dot’s color indicates the −log_10_ *P* value of the enrichment.

Using gas chromatography-mass spectrometry (GC-MS) and liquid chromatography-mass spectrometry (LC-MS) approaches, we identified 66 plasma metabolites differentiating adult and elderly macaques (by differential metabolite screening criteria, variable importance in projection [VIP] value of >1, *P* < 0.05) (Fig. 5A; Table S4). A total of 40 metabolites (including indole, hydroxyproline, and leucine) were significantly higher in adult macaques than the elderly group, and 26 metabolites (including 3-methoxy-4-hydroxyphenylglycol sulfate, maleic acid, and *N*-methyl-2-pyridone-5-carboxamide) were significantly increased in elderly macaques. Pathway enrichment analysis of differential plasma metabolites between adult and elderly macaques showed that glycine, serine, and threonine metabolism (−log_10_ *P* value, 3.9581), Arginine and proline metabolism (−log_10_ *P* value, 3.3471), and starch and sucrose metabolism (−log_10_, *P* value, 3.7115) were significantly enriched. β-Alanine metabolism, synthesis and degradation of ketone bodies and alanine, aspartate, and glutamate metabolism had the largest pathway impact (0.08236, 0.7, and 0.17664, respectively) according to topology analysis (Fig. 5B to 5D).

10.1128/msystems.01512-21.6 TABLE S4 Summary of 66 plasma differential metabolites. Download Table S4, XLSX file, 0.02 MB. Copyright © 2022 Sang et al. 2022 Sang et al. https://creativecommons.org/licenses/by/4.0/ This content is distributed under the terms of the Creative Commons Attribution 4.0 International license.

### Shift of metabolome-microbiome cross talk during aging of macaques.

**FIG 6:**
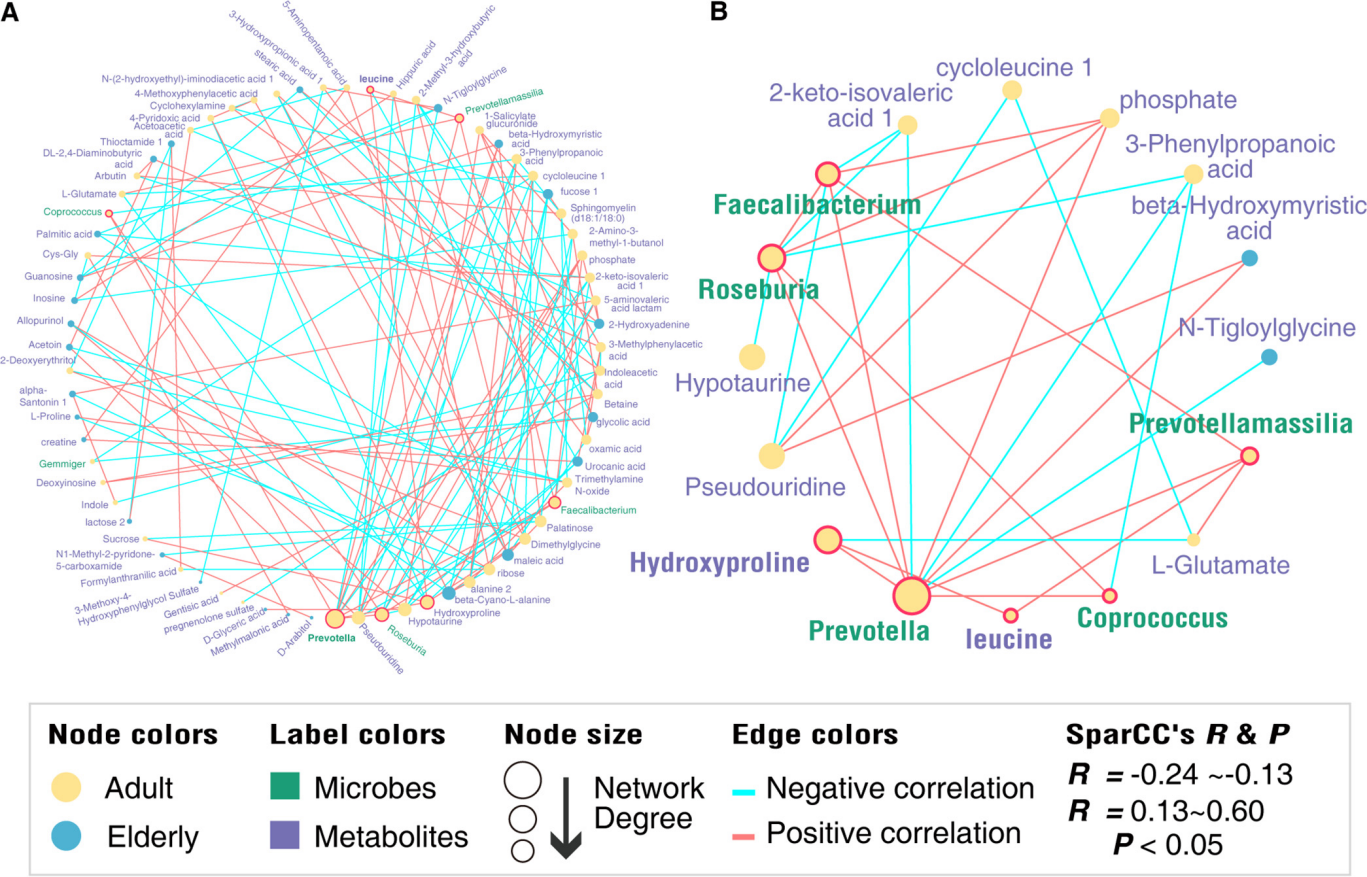
Microbiome-metabolome cross talk during macaque aging (linked to Fig. 3 and 5). (A) Overall co-occurrence network of six common bacterial indicator genera (green labels) and differential metabolites (purple labels) during aging of the macaque. Nodes significantly enriched in adult and elderly macaques are marked in yellow and blue, respectively. Edges with pink and blue indicate significantly positive and negative correlations, respectively. The distribution of network degrees corresponds to node sizes. The SparCC method was used to perform correlation analysis, and the co-occurrence network was visualized by Cytoscape v3.9.1 (84). (B) Metabolites that demonstrated positive interactions directly with five indicator bacterial genera.

Metabolic activities of the gut microbiota play essential roles in the maintenance of host homoeostasis and health. Thus, to estimate effects of aging-related gut microbiota on host metabolism, we further investigated associations between six common indicator bacterial genera (Fig. 3) and 66 differential plasma metabolites (Fig. 5A) during macaque aging. The co-occurrence network reconstructed by SparCC correlation analysis (Table S5 and S6) included 74 positively correlated pairs and 63 negatively correlated pairs, indicating age-related complex interactions between microbiome and microbiome and/or metabolome and microbiome in the macaque (Fig. 6A). We found that the *Prevotella* has the biggest network degree in the network and was significantly and positively correlated with *Faecalibacterium*, *Coprococcus*, *Prevotellamassilia*, and *Roseburia* (Fig. 6B). Interestingly, as a major component of the protein collagen (27), hydroxyproline had the biggest network degree among metabolites (Fig. 6A) and was about 100% more abundant in the plasma of adult than elderly macaques (Fig. 5A). The change with aging in hydroxyproline was significantly positively correlated with changes in abundance of *Prevotella* (Fig. 6B). Leucine, which was enriched in adult macaques, the only differential metabolite in this study consistent with the reported human blood metabolome study (28), was also significantly positively correlated with *Prevotellamassilia* (Fig. 6B).

**FIG 7:**
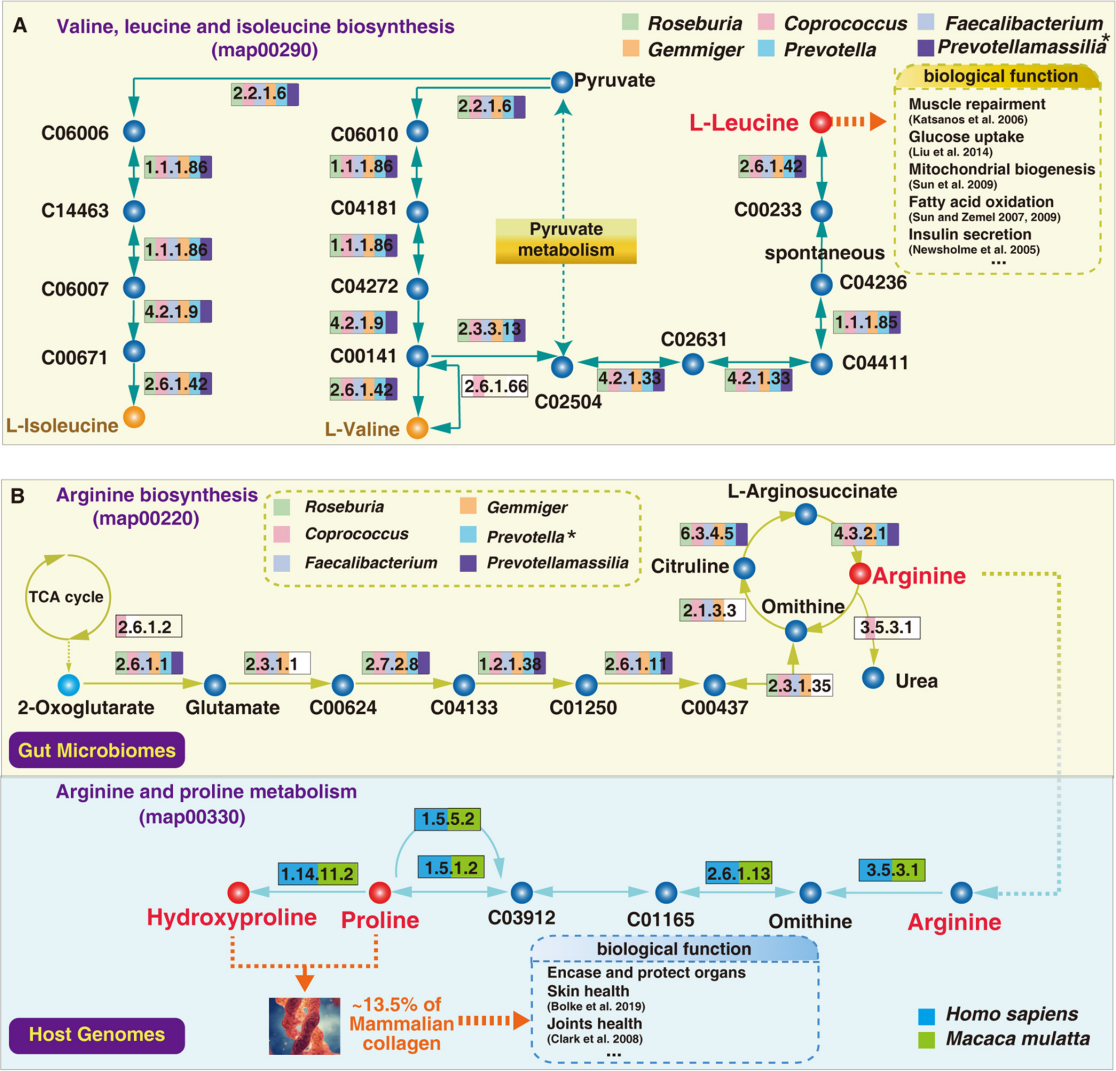
KEGG-predicted abilities to synthesize arginine, proline, hydroxyproline, and leucine of the six indicator genera and their macaque and human hosts. (A) Abilities of these gut microbes to synthesize leucine via the “Valine, leucine and isoleucine biosynthesis” pathway. The asterisk on *Prevotellamassilia* indicates a positive correlation between *Prevotellamassilia* and leucine. (B) Abilities of *Gemmiger*, *Prevotella*, *Faecalibacterium*, *Coprococcus*, *Prevotellamassilia*, and *Roseburia* components of gut microbiomes and host genomes to synthesize arginine, produce hydroxyproline and proline, and metabolize arginine and proline. The asterisk on *Prevotella* represents a positive correlation between *Prevotella* and hydroxyproline.

Corroborating the possibility that changes in hydroxyproline and leucine plasma contents were mediated by changes in abundance of these indicator genera, KEGG predictions showed that these indicator genera have the potential ability to synthesize arginine and leucine (Fig. 7). Leucine can be produced from pyruvate via multistep reactions (Fig. 7A). Arginine can be synthesized from 2-oxoglutarate produced through the tricarboxylic acid (TCA) cycle. KEGG prediction showed that *Prevotella* and *Prevotellamassilia* lack the ability to synthesize three of the enzymes (*N*-acetylglutamate synthase [EC 2.3.1.1], glutamate *N*-acetyltransferase [EC 2.3.1.35], and ornithine carbamoyltransferase [EC 2.1.3.3]) in the arginine biosynthesis pathway, but these two genera had the ability to synthesize argininosuccinate lyase (EC:4.3.2.1) to produce arginine. In addition, the host (human or macaque) can use it to produce hydroxyproline and proline, which are key components of mammalian collagen (Fig. 7B). Thus, these findings indicate that aging-related perturbations of metabolic homeostasis may be strongly linked to shifts in the complex interactions between gut microbes.

## DISCUSSION

We for the first time elucidated the divergence and convergence of aging-related gut microbiota between humans and nonhuman primates. We found that differences in host background, such as genetic and/or dietary factors, are responsible for major differences in these respects between humans and nonhuman primates. However, we also identified six common antiaging gut microbial markers (*Gemmiger*, *Prevotella*, *Prevotellamassilia*, *Roseburia*, *Coprococcus*, and *Faecalibacterium*). Analysis of the macaques’ plasma contents and gut microbiota also indicated that shifts in metabolome-microbiome cross talk may strongly affect the aging process. These results corroborate the benefits of countering aging-associated intestinal dysbiosis and provide a rationale for microbiome-based interventions against age-related diseases using the macaque as a nonhuman primate model.

### Factors affecting variations of gut microbiota in human and nonhuman primates.

Multiple factors affect the formation and stability of the human gut microbiome, including host genotype, lifestyle, diet, and age (29). Numerous pieces of evidence suggest that various dietary styles are the major impacts on human gut microbial variations. For example, it is well known that the compositions of gut microbiota of nonwesternized populations and westernized populations were significantly different, and nonwesternized populations had more new bacterial species (30), largely due to the difference between westernized (high-fat or/and high-protein) and traditional (low-fat and high-carbohydrate) diets. Three enterotypes of the human gut microbiome, respectively, dominated by *Bacteroides*, *Prevotella* and *Ruminococcus*, linked to different diets, have also been identified (31). Westernized populations mostly had the *Bacteroides* enterotypes, and the *Prevotella*-dominated enterotype was most prevalent (95.4% incidence) in nonwesternized people with a healthy lifestyle (32). The populations in Southeast Asian countries (like to eat rice and steamed buns) had and *Ruminococcus* enterotypes (33). For nonhuman primates, we found the captive macaque showed similar gut microbial communities to humans but not chimpanzees or lemurs (Fig. 1D), which cannot parallel the phylogenies of humans and other primates (34) that might have resulted from humanization. This was further confirmed by significant alternations of two dominant phyla, *Firmicutes* and *Bacteroidetes*, among four human populations and three primates (see Fig. S2B in the supplemental material). Interestingly, we found that *Prevotella* was the most abundant bacterial genera in three nonhuman primates (Table S7) and it has the highest abundance in macaques (Fig. S2C). The prevalence of *Prevotella* in the macaque gut is consistent with expectations as it is reportedly more abundant in the gut microbiota of wild macaques than humans (35). These studies suggest that the *Prevotella*-dominated enterotype is the most common in nonhuman primates and that only some humans share a *Prevotella*-dominated enterotype with nonhuman primates. With surprise, we found it consistently significant that the highest levels of *Firmicutes* and the lowest levels of *Bacteroidetes* were in the guts of both the Japanese and Italian populations (Fig. S2B). Accordingly, we found that *Blautia* belonging to the *Firmicutes* phylum, which is linked to traditional and healthy diets (36), is the most abundant genus in Japanese and Italian populations (Table S7). Japanese people have typical healthy eating habits (37), and we found that *Bifidobacterium* is more abundant in the composition of Japanese gut microbiota (Table S7). Our studied Italian population is long-lived, and the gut microbiota of the semisupercentenarians from this population showed significant enrichment of *Bifidobacterium* (18). These findings suggest it is very important to understand the role of gut microbiota-diet interaction in healthy aging. Although our studied Chinese people is also long-lived (19), this study indicated the Chinese people may be facing the negative impacts from westernized diets because the Chinese and British subjects have more similar gut microbial communities (Fig. 1D) and dominant gut microbiota composition (Table S7) than other humans or nonhuman primates. For example, these two populations have the most abundant gut bacterial genus *Bacteroides* (Fig. 2C) that can signal high-fat or/and high-protein dietary style (31). Consequently, it is imperative to optimize dietary styles to promote healthy aging of Chinese people in the future.

10.1128/msystems.01512-21.2 FIG S2 Relative abundances of all 22 gut bacterial phyla (A), the three dominant phyla *Firmicutes*, *Bacteroidetes*, and *Actinobacteria* (B), and the three genera *Bacteroides*, *Prevotella*, and *Ruminococcus* (C) across all subjects studied. Download FIG S2, EPS file, 2.7 MB. Copyright © 2022 Sang et al. 2022 Sang et al. https://creativecommons.org/licenses/by/4.0/ This content is distributed under the terms of the Creative Commons Attribution 4.0 International license.

10.1128/msystems.01512-21.7 TABLE S5 Relative abundance of the six indicator genera and 66 plasma differential metabolites in the macaques. Download Table S5, XLSX file, 0.04 MB. Copyright © 2022 Sang et al. 2022 Sang et al. https://creativecommons.org/licenses/by/4.0/ This content is distributed under the terms of the Creative Commons Attribution 4.0 International license.

10.1128/msystems.01512-21.8 TABLE S6 SparCC correlation analysis of the six indicator genera and 66 plasma differential metabolites in macaques. Download Table S6, XLSX file, 0.01 MB. Copyright © 2022 Sang et al. 2022 Sang et al. https://creativecommons.org/licenses/by/4.0/ This content is distributed under the terms of the Creative Commons Attribution 4.0 International license.

10.1128/msystems.01512-21.9 TABLE S7 Summary of the relative abundance of all gut bacterial genera in seven host groups. Download Table S7, XLSX file, 1.2 MB. Copyright © 2022 Sang et al. 2022 Sang et al. https://creativecommons.org/licenses/by/4.0/ This content is distributed under the terms of the Creative Commons Attribution 4.0 International license.

Other accumulated evidence has also demonstrated the impacts of host gut microbiomes. For example, the effects of heritability and host genetics on human gut microbiota and metabolic syndrome have been discovered (38). A recent study based on 585 yellow baboons sampled over 14 years further corroborates the partial heritability of most gut microbiota traits (39, 40). The sympatric chimpanzees and gorillas harbor convergent gut microbial communities (41). The deletion of 2.3 kb in the *N*-acetyl-galactosaminyl-transferase gene was recently confirmed to determine the decreasing of *Erysipelotrichaceae* abundance in the intestine of pigs (42). In short, the strong influence of diet and host genetics on gut microbiota might explain high variations of aging-associated gut microbial signals across different humans or nonhuman primates observed in this study and other known reports (7).

### Butanoate-producing bacteria as candidate antiaging markers.

Despite high variations in gut microbial diversities of humans and nonhuman primates, we identified six common indicator genera (*Prevotella*, *Prevotellamassilia*, *Coprococcus*, *Roseburia*, *Faecalibacterium*, and *Gemmiger*) from the gut microbiotas of seven host groups (Fig. 3; Table S2). These bacteria have the potentials as antiaging markers because these bacteria have predicted abilities to produce butanoate through butyrate kinase and phosphate butyryltransferase (Fig. 4). Many previous studies also partially supported our observations. Compared to the *Bacteroides* enterotype, the *Prevotella* enterotype could produce more short-chain fatty acids (43). Experimental evidence suggested that isolated strains of Faecalibacterium prausnitzii (A2-165 and L2-6) and Roseburia intestinalis can produce butyrate (44, 45). Additionally, the reduction of butyric acid-producing bacteria was frequently associated with the presence of various human diseases. For example, the Faecalibacterium prausnitzii and *Roseburia*, are reduced in intestines of patients with ulcerative colitis (46). The relative abundance of *Faecalibacterium*, *Roseburia*, *Clostridium sensu stricto*, *Gemmiger*, *Dialister*, *Romboutsia*, *Coprococcus*, and *Butyricicoccus* was decreased in AD patients (47). *Roseburia* and *Faecalibacterium* are also reduced in patients with Parkinson's disease (48). Overall, these studies suggest that butyric acid-producing bacteria play important roles in their host’s health. Our identified six common indicator bacterial genera might modulate the aging process by the anti-inflammatory effect of butyric acid (49).

### Antiaging plasma metabolite markers.

One previous human study suggested that blood leucine was significantly enriched in younger compared to elderly people (28), consistent with our finding (Fig. 5A). Leucine supplements in the diet can ameliorate response of muscle protein synthesis in the elderly (50). Based on the functional predictions of KEGG, our results discovered the abilities of leucine synthesis of all six common indicators (Fig. 7A), indirectly supporting our metabolic profiling of leucine in macaque plasma (Fig. 5A). Such findings also indicated that six common indicator bacterial genera may ameliorate diverse health problems associated with leucine deficiency during human aging.

We also found that hydroxyproline, which accounts for roughly 13.5% of mammalian collagen (51), was enriched in plasma metabolites of adult macaques (Fig. 5A). Accordingly, the reported decline in hydroxyproline in serum metabolites with human aging suggests that hydroxyproline is an important aging-related marker in blood (52). This raises the question of whether there is a direct or indirect relationship between hydroxyproline enrichment in young macaques and their gut microbes. The KEGG pangenomic analysis in this study shows that six common indicators can synthesize arginine, and both human and macaque hosts can utilize it (Fig. 7B). Hydroxyproline and proline play key roles in maintenance of collagen stability (27), and we found that both the host (human and macaque) and gut microbiota participate in their synthesis (Fig. 7B). As collagen plays important roles, for example, in encasing and protecting organs, provision of supplemental collagen is generally considered to be an effective approach for ameliorating skin aging (53) and improving joint health (54). Such healthy effects might be associated with the interplay between specific gut functional bacteria and their hosts.

Other plasma metabolites enriched in adult macaques discovered by this study, including indole, inosine, hypotaurine, and betaine, have potential antiaging effects in various ways in diverse model animals and humans. For example, indole from symbiotic microflora has been found to prolong the healthy life span of various organisms, such as Caenorhabditis elegans, Drosophila melanogaster, and mice (55). Inosine also has anti-inflammatory effects in many types of human cells, such as monocytes, neutrophils, and epithelial cells, thus protecting alveolar epithelial cells from DNA damage induced by hyperoxia (56, 57). Hypotaurine can be used to enhance oxidative stress resistance and promote longevity in Caenorhabditis elegans (58). Chronic and low-grade inflammation are hallmarks of human aging (59), betaine can relieve chronic inflammation by inhibiting the NF-κB signaling pathway (60) and low betaine concentrations in humans increases risks of cardiovascular disease (61). These results clearly indicate the roles of gut microbiota and their derived metabolites during host healthy aging.

### Implications for macaque as a model and future gut microbial studies.

We found that captive macaques showed similar gut microbial communities to Chinese subjects (Fig. 1D), and five of the six common indicator genera were found in macaques (Fig. 3; Table S2). In addition, the macaque is genetically related to humans, with 92.5 to 95% genetic homology (62). These findings suggested that the macaques can be used as optimal animal model to study the effects of gut microbiota on healthy aging or human aging-related diseases. However, our study found that *Prevotella* is the common dominant in nonhuman primates, but the *Bacteroides* are much more abundant in humans (Fig. S2C), indicating distinct differences in gut core bacteria between humans and nonhuman primates. Thus, it is of considerable interest how to build humanized animal models to study effects of gut microbiota on human health. Here, we provide several proposals for use of the macaque as an animal model in the study of gut microbiota. The first important thing to reconstruct is the humanized gut microbiota in macaques by simulating dietary styles or habits of specific human populations before starting experiments. Another concern is to accelerate humanized progress of gut microbiota by constructing genetically humanized monkeys with scientific estimates of health risks via transgenic tools. Finally, it is necessary to keep gender and age consistent in experimental designs.

### Conclusions.

We found that *Coprococcus*, *Roseburia*, *Gemmiger*, *Prevotella*, *Prevotellamassilia*, and *Faecalibacterium* are candidate antiaging markers for both humans and nonhuman primates. These indicator bacteria potentially contribute to the production of pivotal metabolites such as butyrate, leucine, and hydroxyproline, which are beneficial for healthy aging in humans and nonhuman primates, although further functional evidence is needed. Our findings also provide valuable foundations for using the macaque as an animal model to study the gut microbiome’s role in human health and disease.

## MATERIALS AND METHODS

### Ethics approval on this research.

All healthy rhesus macaques used in this study were of Chinese origin and raised in the Kunming Primate Research Center, Kunming Institute of Zoology, Chinese Academy of Sciences, Kunming, China. All animal procedures were conducted following the international standards and were approved in advance by the Institutional Animal Care and Use Committee of Kunming Institute of Zoology, Chinese Academy of Sciences. All monkeys were given commercial monkey biscuits twice a day with tap water *ad libitum* and were fed fruits and vegetables once daily. All animals selected for this study were healthy and had normal food intake. They had no clinical treatments and did not take any antibiotics within a month before starting the experiment.

### Sample collection.

Because a year for a macaque is approximately equivalent to 3 years for a human (63), we raised two groups of individuals: 18 “young” and 31 “elderly” macaques (matched human ages of 12 to ~15 [mean ± SD, 13.5 ± 1.5] and 42 to ~78 [60 ± 9.53] years, respectively) in separate cages. Fresh fecal and corresponding blood samples of the macaques were collected in August 2018 and stored at −80°C until further processing. Detailed information of all macaques is provided in Table S1 in the supplemental material.

### Bacterial DNA extraction, amplification, and sequencing.

Bacterial genome DNA was extracted using a QIAmp DNA stool minikit (Qiagen, Inc.; catalog no. 51604) following the manufacturer’s instructions. The completeness of DNA templates was checked by agarose gel electrophoresis, and the concentration was quantified with a NanoDrop ND-1000 spectrophotometer. The V3-V4 hypervariable region of the 16S rRNA gene was amplified using the universal bacterial primers 341F (5′-ACTCCTACGGGAGGCAGCA-3′) and 806R (5′-GGACTACHVGGGTWTCTAAT-3′) (64). The target sequences were amplified in 25-μL reaction mixtures. The amplification conditions consisted of 5 min at 95°C, 30 cycles of 1 min at 95°C, 1 min at 50°C, and 1 min at 72°C followed by a final 7-min step at 72°C. After purification, the amplicons were sequenced on an Illumina MiSeq platform in the 300-bp paired-end mode.

### Data citation.

To obtaining general age patterning of humans and nonhuman primates, we first downloaded 16S rRNA gene data sets from four healthy human populations, including 26 Italians (18), 88 Chinese (19), 208 Japanese (20) and 64 British individuals (American Gut Project; http://americangut.org). The age ranges for adults and the elderly were set to 20 to 40 years old and 50 to 80 years old, respectively. We also obtained 16S rRNA gene data sets of gut microbiota from another two nonhuman primate species, including 451 individuals of Pan troglodytes (chimpanzee) (22) and 26 individuals from Lemur catta (21). The age of lemur matching to human was estimated based on the AnAge database of animal aging and longevity (https://genomics.senescence.info/species/biblio.php?id=29). The matched human age of chimpanzee is approximately equivalent to 3 or 1.5 years (65). All detailed information from all studied subjects is provided in Table S1.

### Bioinformatic analysis of 16S rRNA gene sequences.

The overall analysis of 16S rRNA data was based on the QIIME1, QIIME2, USEARCH (version 11.0.667 i86linux64), and MOTHUR pipelines (66–69). The paired-end reads were merged by the “usearch -fastq_mergepair” command with up to 1 expected error per tag. Single-end reads used a quality filter by the fastp software with default parameters (70). All the merged paired-end reads and single-end reads were converted to fasta with iTools as previously described (71). The fasta file obtained in the previous step was aligned with the SILVA database (v138) (72) by MOTHUR to trim out the V4 region of the 16S rRNA. The commands “usearch11_x64 - fastx_uniques” and “usearch11_x64 -sortbysize” were used to identify and delete unique sequences. The “usearch11_x64 -unoise3” command was applied for denoising and acquisition of ZOTUs. The “usearch11_x64 -sintax” command was then applied for ZOTU taxonomic prediction using the RDP training set v18 (73, 74). The “usearch11_x64 -otutab_trim” command was applied to remove low-abundance ZOTUs and samples with few reads (at least 6 reads for ZOTUs and 100 reads for each sample, respectively). Next, the “alpha_diversity.py” and “beta_diversity.py” commands were used to calculate alpha diversity indices and beta distance matrixes, respectively. We subsampled all sequences to 1347 tags per sample to keep many more lemur samples according to the minimum number of tags in lemur samples.

### Functional predictions.

We predicted potential functions of both indicator bacterial genera according to genus-level pangenome analysis using the KEGG genome database (https://www.genome.jp/kegg/genome/). Similarly, we also predicted animal hosts’ abilities to utilize arginine by pangenome mapping of the pathway “Arginine and proline metabolism.” For the given bacterial genus without the annotations of the KEGG database, we searched nonredundant protein (nr) annotation of the bacterial genus in NCBI (https://www.ncbi.nlm.nih.gov/) using four key enzymes involved in butyrate production, including *trans*-2-enoyl-CoA reductase (NAD^+^; EC:1.3.1.44), butyrate kinase (EC:2.7.2.7), acetate CoA-transferase (EC:2.8.3.8), and phosphate butyryltransferase (EC:2.3.1.19) (referring to the butyrate metabolism pathway map00650 in KEGG). The obtained enzyme sequences were BLAST searched against the nr database again to confirm the reliabilities of targeted enzymes (see the results in Table S3) under the thresholds of alignment coverage higher than 90%, E value lower than 1e−5 and at least 30% identity. Finally, predicted results were used to determine whether the indicator bacteria have potentials for butyrate production (Fig. 4).

### Macaque plasma metabolomic profiling.

Macaque plasma metabolites were detected with ultrahigh-pressure liquid chromatography (UHPLC)-quantitative time of flight mass spectrometry (here, LC-MS/MS) and gas chromatography-time of flight-mass spectrometry (here GC-TOFMS).

For the GC-TOFMS analysis, we used an Agilent 7890 gas chromatograph system combined with a Pegasus HT time-of-flight mass spectrometer (J&W Scientific, Folsom, CA, USA). ChromaTOF 4.3X software (LECO Corp.) and the LECO-Fiehn Rtx5 database were used for extraction of raw peaks, baseline filtering and calibration, peak alignment, deconvolution, peak identification, and integration of peak areas (75). The retention time index (RI) method was used for peak identification, and the RI tolerance was 5,000. Macaque plasma metabolite peaks that were present in <50% of quality control (QC) samples were removed (76). In chromatograms of the initial set of samples, which included 49 experimental samples and eight QC samples, 448 effective peaks were identified. To analyze the data, we first removed noise spikes and outliers based on the interquartile range. We then removed peaks with more than 50% null values from the data sets for either the adult or elderly macaques. Next, missing peak values were recorded as half the minimum recorded values. Finally, internal standards were used for normalization.

Liquid chromatographic analysis was performed using a UHPLC system (1290, Agilent Technologies) equipped with an Acquity UHPLC BEH amide column (1.7 μm, 2.1 by 100 mm; Waters) coupled to a TripleTOF 6600 (AB Sciex) quadrupole time of flight (Q-TOF) instrument. The mobile phase consisted of 25 mM NH_4_OAc and 25 mM NH_4_OH in water (pH 9.75) (phase A) and acetonitrile (phase B). The flow rate was 0.3 mL/min, and the proportion of phase B linearly declined from 85% to 75% from 0 to 2 min and then to 0% between 2 and 9 min. Following a hold at 0% phase B until 14 min, the proportion of phase B was returned to 85% between 14 and 15 min, and the column was reequilibrated with 85% phase B for a further 5 min before the next injection. The linked TripleTOF mass spectrometer was operated in both positive- and negative-ion modes (77). Analyst TF 1.7, AB Sciex acquisition software continuously evaluated the full-scan MS data, triggering MS/MS spectrum acquisition according to preselected criteria. In each cycle, six precursor ions with an intensity greater than 100 were selected for fragmentation at 35-V collision energy (CE) (15 MS/MS events with a 50-ms product ion accumulation time). The electrospray ionization (ESI) source settings were as follows: ion source gas 1 and ion source gas 2; curtain gas pressures of 60, 60, and 30 lb/in^2^, respectively; source temperature of 550°C; and ion spray voltage floating (ISVF) values of 5,500 and −4,500 V in positive and negative modes, respectively.

In total, 1,760 features were identified in the 49 experimental samples. We then processed the data by removing noise spikes and outliers, based on the relative standard deviation (RSD), recorded missing values as described above, and used the total ion current (TIC) for each sample for data normalization.

For both LC-MS/MS and GC-TOFMS data, we applied Student's *t* test (set at *P* < 0.05) and variable importance in projection (VIP) values of >1.0 to identify metabolites with significantly different plasma levels in the young and elderly macaques. We identified 66 differential metabolites (Table S4). For every single metabolite detected by both models, the abundance of the metabolite in the model with a smaller *P* value by *t* test was chose. Differential metabolites were mapped in the KEGG database (www.kegg.jp/kegg/pathway.html) to detect differentially expressed pathways. The enrichment pathways’ impact of differential metabolites was obtained by topological analysis (78).

### Statistical analysis.

Bray-Curtis distances of microbial communities across samples were used to perform PCoA analysis in the “vegan” package in R (version 4.1.3) and visualized using the ggplot2 package (79, 80). For PCoA analysis, adjusted *R*^2^ and *P* values were calculated by the functions “RsquareAdj” and “adonis,” respectively, in the “vegan” packages in R. The UPGMA tree of microbial communities was also built using “ phangorn” package (81) in R based on Bray-Curtis distances. To analyze the domain taxonomy of each group, we used the indicator value algorithm to analyze the classification of genus level (26, 82). Indicator values were calculated using the “indval” function in the “labdsv” package in R (version 4.1.3) (26).

Wilcoxon's rank-sum test in R was employed to test significant difference between groups. The SparCC method (83) was used to perform the correlation analysis between six shared indicator genera and plasma metabolites of the macaque, with *P* values of <0.05 statistically significant. The co-occurrence network between microbiomes and metabolomes was built and visualized using Cytoscape (v3.9.1) (84).

### Data availability.

The raw sequence data from macaques reported in this article were deposited in the Genome Sequence Archive (85) in the National Genomics Data Center (86), China National Center for Bioinformation/Beijing Institute of Genomics, Chinese Academy of Sciences, under accession number CRA004103 and are publicly accessible at https://bigd.big.ac.cn/gsa.
